# A review of the efficacy of clinical tuberculosis vaccine candidates in mouse models

**DOI:** 10.3389/fimmu.2025.1609136

**Published:** 2025-05-29

**Authors:** Lidia del Pozo-Ramos, Andreas Kupz

**Affiliations:** Australian Institute of Tropical Health and Medicine, James Cook University, Cairns, QLD, Australia

**Keywords:** BCG, tuberculosis, vaccine, clinical, mouse models, host genetics

## Abstract

Tuberculosis (TB) remains one of the deadliest infectious diseases worldwide, causing over a million deaths annually. The only licensed TB vaccine for human use, Bacille Calmette-Guérin (BCG), a mycobacteria-based live-attenuated vaccine, confers immunity to children but fails to efficiently protect adults from pulmonary TB. Several TB vaccine candidates have been developed over the last two decades, but some have failed to provide substantially better protection than BCG in clinical trials. Most of these vaccine candidates were initially evaluated for their protective capacity in mouse models of TB. With the availability of several mouse strains, vaccination routes and *Mycobacterium tuberculosis* (*Mtb*) challenge strains, to-date there is no consensus in the field about the predictive value of different murine models of TB, and it remains a matter of debate whether host genetics or vaccine-driven parameters primarily determine vaccine efficacy. Here we reviewed the performance of all TB vaccine candidates that have entered clinical trials over the last 25 years. We extracted protective efficacy data from all published studies that utilized mouse models to assess vaccination efficacy. The efficacy of each vaccine candidate to reduce lung bacterial burden depending on the mouse genotype, the vaccine administration route, and the *Mtb* challenge strain at different time-points was evaluated. Our data reveals insights into the effect of experimental parameters on vaccine performance and emphasizes the potential benefits of standardizing TB mouse models across vaccination-challenge studies to identify pre-clinical vaccine candidates with the highest potential to succeed.

## Introduction

Tuberculosis (TB) remains a serious global health challenge with about twenty-five per cent of the worldwide population being infected with *Mycobacterium tuberculosis* (*Mtb*), the causative bacterial agent of TB, at some stage in their life (1, 2). According to the World Health Organization (WHO), it has been estimated that in 2023, 10.8 million people fell ill and 1.3 million died from TB worldwide (1). *Mtb* disperses easily through the air in aerosols when a person with an active manifestation of TB coughs (3), sneezes and/or spits (4). While TB often affects the lungs as pulmonary disease, it can disseminate to nearly all organs in the body causing extrapulmonary manifestations. In most cases *Mtb* is contained by the immune system in a state of latent, asymptomatic infection (5). However, about 5 to 10% of people with latent TB will progress to active TB over time and more than half of those cases will die if they do not obtain efficient treatment (1, 6).

The United Nations Sustainable Development Goals have set the priority health target of ending the TB epidemic by 2030 (7). To achieve that target, prevention of further cases is fundamental, which involves early screening and detection of those at high risk, facilitated by developments of new diagnostic tools, treatment of cases, reducing reservoirs of latent TB, and vaccination (4). Notwithstanding the availability of treatment, the rise in newly diagnosed TB cases to 7.5 million in 2022, the highest number since the WHO began monitoring in 1995, and the increasing rates of multi-drug resistant (MDR)-TB, effective vaccines are considered the most important control method (8).

Bacille Calmette Guérin (BCG), a live-attenuated vaccine (LAV) developed by Albert Calmette and Camille Guérin, remains currently the only WHO-approved and licensed TB vaccine for human use since its first use in 1921. BCG is an attenuated strain of *Mycobacterium bovis (M. bovis)*, the causative agent of TB in cattle. For its attenuation, *M. bovis* was sub-cultured for 13 years, in the process likely losing more than 100 genes (9). Although BCG prevents extrapulmonary forms of TB in children (the reason why it is still used in millions of infants today), it is not effective against all TB disease manifestations in all populations, particularly pulmonary TB in adolescents and adults (10). The efficacy of BCG depends on several factors, including the strain of BCG (there are multiple licensed strains of BCG derived from the original strain obtained by Calmette and Guérin) the host immune system, the existence of any comorbidities and host genetics (11). Of note, in 2018 the WHO declared that BCG was inadequate as a therapeutic vaccine, as preexposure to *Mtb* or contact with other environmental mycobacteria negatively affect the efficacy of the vaccine (12). Furthermore, BCG is currently administered intradermally (ID) in humans; however, it is known that this route provides also insufficient protection in animals models. Although intradermal vaccination induces systemic T cell responses, insufficient levels of airway luminal T cells, antibody and lung resident memory T cells are generated by this route to provide a long-lasting protection (13). Hence, the evaluation of intratracheal (IT) or intranasal (IN) or intravenous (IV) (14) administered BCG remains an area of active research (15).

**Figure 1:**
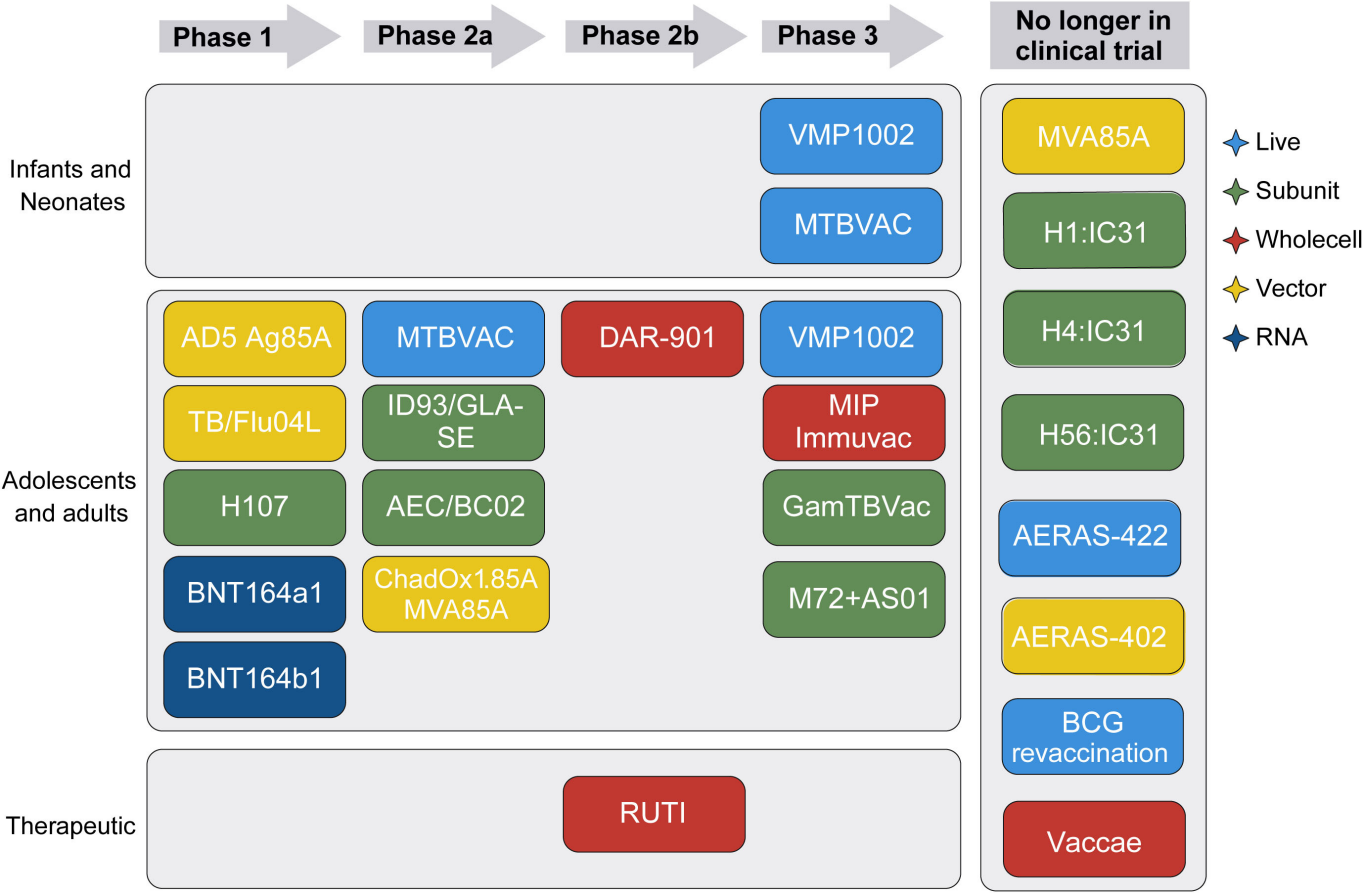
TB vaccine candidates in the clinical trial pipeline. Schematic showing the developmental stages of various TB vaccine candidates, depending on target population, clinical trial phase, and vaccine type. Vaccine type is indicated by color. Modified from the Tuberculosis Vaccine Initiative TB Vaccine Pipeline, last updated October 2024 (22).

To improve or replace BCG, over the last 30 years, different types of TB vaccine candidates have been and are currently being developed. They include LAVs, subunit and viral-vectored vaccines as well as whole cell inactivated bacteria. Moreover, new technologies such as mRNA (16, 17), coated spores (18), bacterial ghosts (19), peptides (20) and outer bacterial membranes (21) have been used as vaccine platforms. Since 2002, several of those TB vaccine candidates have entered clinical trials, but unfortunately several have failed to provide better protection than BCG in efficacy trials (**Figure 1**) (12, 23).

Overall, the development of improved vaccines against *Mtb* has proven to be challenging for two main reasons:

Firstly, because the immune responses correlating with protection against TB are still largely unclear, it remains difficult to develop rationally designed subunit vaccines. It also impacts the selection of end points for clinical trials, in which efficacy is measured against BCG. Currently clinical efficacy trials require a very high number of participants and long-term follow-up, making clinical studies lengthy and expensive. Elucidating the correlates of protection against TB in humans will significantly advance future vaccine development. The second critical reason for the *‘failure’* of some TB vaccine candidates to outperform BCG in clinical trials has been the lack of predictive animal models for pre-clinical evaluation and shortlisting. The use of animal models for TB started in 1865 when Jean-Antoine Villamin demonstrated TB transmission in rabbits (24), and experimental studies in guinea pigs and mice were introduced for TB soon after Robert Koch identified *Mtb* in 1882. Koch demonstrated that inoculation of mice with *Mtb* produced similar lesions to those seen in human TB (25, 26). Subsequently, researchers developed various other animal models for TB, including rats, cattle, goats, zebrafish, and non-human primates (27). While not suitable to study vaccine-mediated protection, invertebrate models of TB have also been established in amoeba and the fruit fly (28).

For many reasons, to-date, mice remain the most common animal model used in TB vaccine research, as they share many physiological and genetic similarities with humans (29), are cost-effective, easy to care for, have a rapid reproduction and life cycle and allow for large group sizes. Immunological responses to *Mtb* are well understood in mice, and there is access to a wide range of immunological and genetic tools for mice (30). However, *Mtb* is not a natural pathogen for mice, resulting in low susceptibility to the bacilli and therefore, mice can often carry significant amounts of *Mtb* without showing major disease symptoms. In addition, the lung pathology that is associated with TB in mice is often different from what is seen in humans (31). Over time, different mouse strains have been evaluated in their response to *Mtb* infection and for their ability to mimic elements of human TB disease. It is now clear that different mouse strains show variations in their susceptibility to *Mtb* with some strains being resistant to lethal infection, while other strains succumb rapidly to even a low dose of *Mtb*. For example, the most common mouse strains used in TB research, BALB/c and C57BL/6, are considered relatively resistant to *Mtb (* 32), while CBA, DBA/2, C3H, 129/S2 (33) mice are much more susceptible and develop more severe disease. This notion of host-genetics as a significant driver of TB susceptibility and disease severity was further supported by results obtained in Collaborative Cross (CC) and Diversity Outbred (DO) mice that show a genetic variation that is typical of outbred populations (34, 35). The founder strains of DO and CC mice (A/J, C57BL/6J, 129S1/SvlmJ, NOD, ShiLtJ, NZO/HILtJ, CAST/EiJ, PWK/PhJ, and WSB/EiJ) together contain 37.8 million single nucleotide polymorphisms (SNPs) along with 6.9 million insertions, deletions, and structural variants. This genetic diversity makes these models extremely valuable for studying complex traits and exploring genotype-phenotype relationships similar to a diverse human population (35, 36). CC strains are a collection of recombinant inbred lines developed from the genetically diverse founder strains. Currently, around 50 CC strains are available for distribution. DO mice originate from the continuous outcrossing of these founder strains, leading to extensive genomic recombination. This process results in high heterozygosity and genetic diversity, making DO mice particularly valuable for high-resolution genetic mapping (37).

While mouse and bacterial genetic diversity is now emerging as an important contributor to vaccine efficacy, including for BCG (38), many of the TB vaccine candidates in the clinical trial pipeline were developed prior to this understanding. Furthermore, there remains debate in the field of which mouse strain(s) should be used to evaluate novel TB vaccine candidates.

To assist with this debate, here we attempted to review the performance of all TB vaccine candidates that have entered clinical trials since 2002. We extracted protective efficacy data from all published studies that utilized mouse models to assess vaccination efficacy. The efficacy of each vaccine candidate to reduce lung bacterial burden depending on the mouse genotype, the vaccine administration route, and the *Mtb* challenge strain at different time-points was evaluated. In parallel, we also extracted data on the efficacy of BCG to lower *Mtb* burden in the lung relative to unvaccinated mice. While great efforts were made to identify all published studies for the clinical vaccine candidates, we apologize to the respective investigators if a particular paper was missed. It is important to note that the purpose of this article is not to compare the performance of individual vaccine candidates against each other, nor to provide any judgement about their potential value as future TB vaccines.

## Materials and methods

### Selection of candidate vaccines

To identify TB vaccines for inclusion into this review, the following criteria were applied:

i) The vaccine was included in the Tuberculosis Vaccine Initiative (TBVI) Vaccine Development Pipeline at some stage until February 2025; ii) Vaccines have been tested in at least one mouse model; iii) The efficacy of the vaccines was compared to BCG in the lung.

### Selection of literature sources

Literature review included the following search criteria: i) articles were written in English; ii) they included the vaccine candidate and/or BCG; and iii) keywords included mouse/mice/*in vivo*/murine. In addition, we focused on studies that reported lung colony forming unit (CFU) data, particularly those providing delta log reductions in CFU in the lungs as a key measure of vaccine efficacy.

### Data extraction

**Figure 2:**
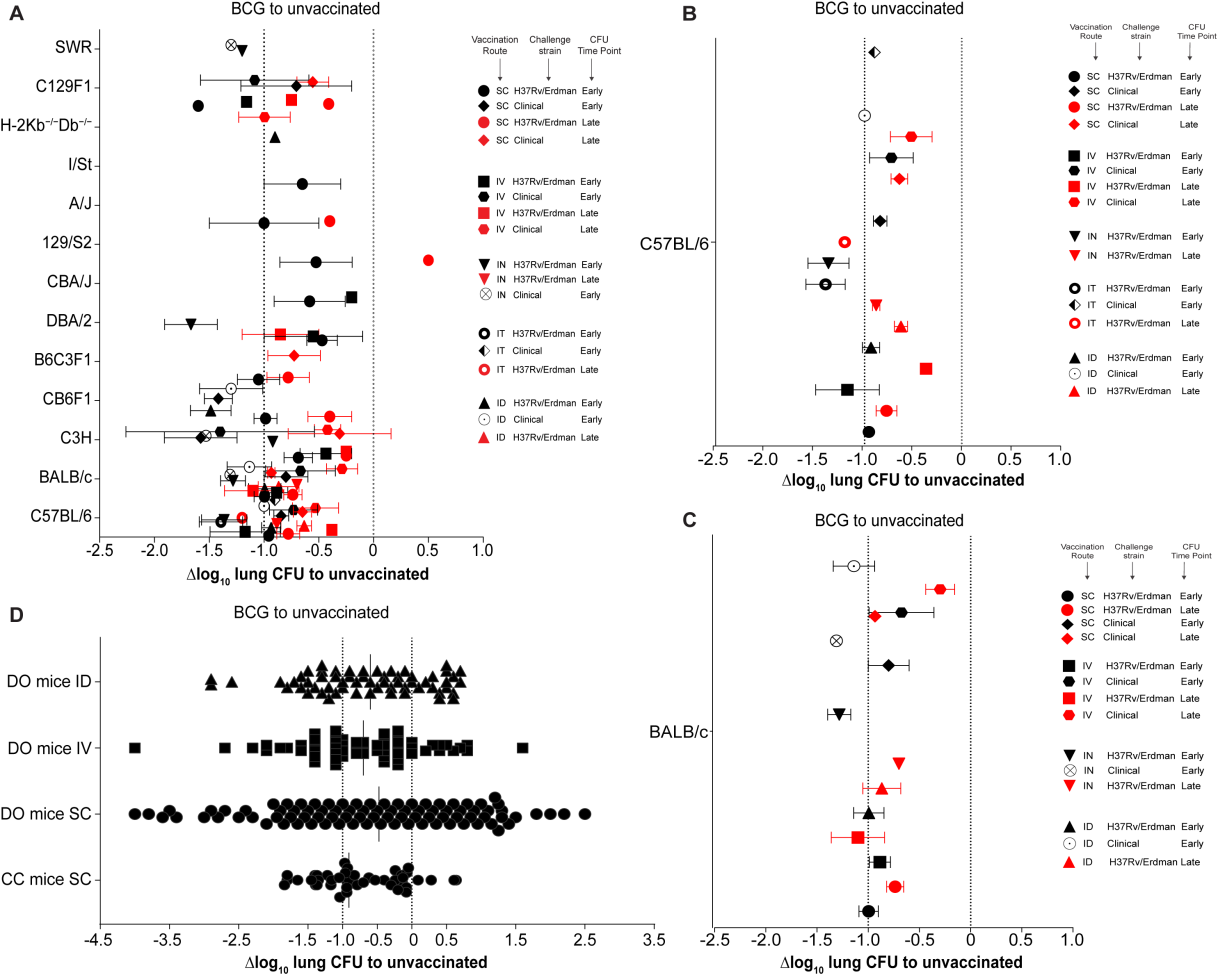
Comparison of BCG efficacy across various mouse models and vaccination routes. **(A)** Δlog_10_ change in lung *Mtb* CFU in various mouse strains compared to unvaccinated controls. Symbol color indicates time point of efficacy measurement post *Mtb* challenge (black: early time points < 60 days post-challenge; red: late time points > 60 days post-challenge). Symbol shape indicates different combinations of mouse strain, route of vaccination, and *Mtb* challenge strain. **(B, C)** Enlargement of results from C57BL/6 **(B)** and BALB/c mice **(C)** shown in **(A)**. **(D)** Δlog_10_ lung bacterial loads in Collaborative Cross (CC) and Diversity Outbred (DO) mice following BCG vaccination. Dotted lines indicate Δlog_10_ reference values of 0 and -1.

If available, raw data was extracted from the manuscript or Supplementary Materials. In cases where no raw data CFU values were available, delta CFU values were extracted from Figures by measuring the log_10_ CFU distance on the y-axis between i) BCG vs. unvaccinated controls; and ii) vaccine candidate vs. BCG. Most of the data for BCG efficacy vs. unvaccinated mice were extracted from the broader search on experimental TB vaccine candidates, ensuring comprehensive coverage of both BCG and novel vaccine candidates in the context of CFU reduction. Additional publications on BCG efficacy were chosen to comprehensively cover the different vaccination routes and time points to measure efficacy. For SC and ID vaccination routes, for which the largest amount of literature exists, additional publications were selected randomly. Due to the very large number of DO mice vaccinated SC by Kurts et al. (39, 40), only one mouse per 0.05 Δlog_10_ CFU increment was plotted for **Figure 2D**. Given the vast literature on BCG efficacy relative to unvaccinated mice for over 100 years, we limited the literature search to 100 publications (**Table 1**; **Supplementary Table S1**).

**Table 1:** Overview of mouse studies performed to evaluate clinical TB vaccine candidates.

Vaccine type	Vaccine candidate	Number of studies	Mouse strains	Vaccination routes	References
Live attenuated	MTBVAC	6	C57BL/6, BALB/c, C3H/HeNRJ	SC, IV, ID, IP, IN, IT	(41–46)
	VMP1002	9	C57BL/6, BALB/c	SC, IV	(47–54)
	AERAS-422	3	C57BL/6	SC	(54–56)
Whole cell	*M. vaccae/*SRL172/ DAR-901	2	C57BL/6, BALB/c	ID, SC	(57, 58)
	MIP-Immuvac	2	C57BL/6, BALB/c, C3H/HeNCrl, CBA/N	SC, Aerosol	(59, 60)
	RUTI	1	C57BL/6	SC	(61)
Subunit	ID93/GLA-SE	8	C57BL/6, H-2Kb^−/−^Db^−/−^	SC, IM, IN	(62–69)
	GamTBVac	1	C57BL/6	IV	(70)
	H1:IC31	7	C57BL/6, BALB/c, CB6F1	SC, IP	(71–78)
	H4:IC31	3	C57BL/6, CB6F1	SC	(72, 79, 80)
	H56:IC31	2	C57BL/6, CB6F1	SC	(76, 81)
	H107	2	CB6F1, B6C3F1	SC	(82, 83)
	M72F/AS01_E_	2	C57BL/6	IM	(84, 85)
Vector	ChadOxMVA85A	2	BALB/c	IN, ID	(86, 87)
	Ad5Ag85A	2	BALB/c	IM, IN, SC	(88, 89)
	MVA85A	4	C57BL/6, BALB/c	IM, ID, IN, SC	(53, 86, 90, 91)
	TB/Flu04L	1	C57BL/6	IN	(92)
	AERAS-402	1	C57BL/6, BALB/c	IM, IN	(93)
Licensed vaccine	BCG	100	C57BL/6, BALB/c, C3H, CB6F1, B6C3F1, DBA/2, CBA/J, 129/S2, A/J, I/St, H-2Kb^−/−^Db^−/−^, C129F1, SWR, CC, DO	SC, IV, IN, IT, ID	See **Supplementary Table S1**

### Data visualization

**Figure 3:**
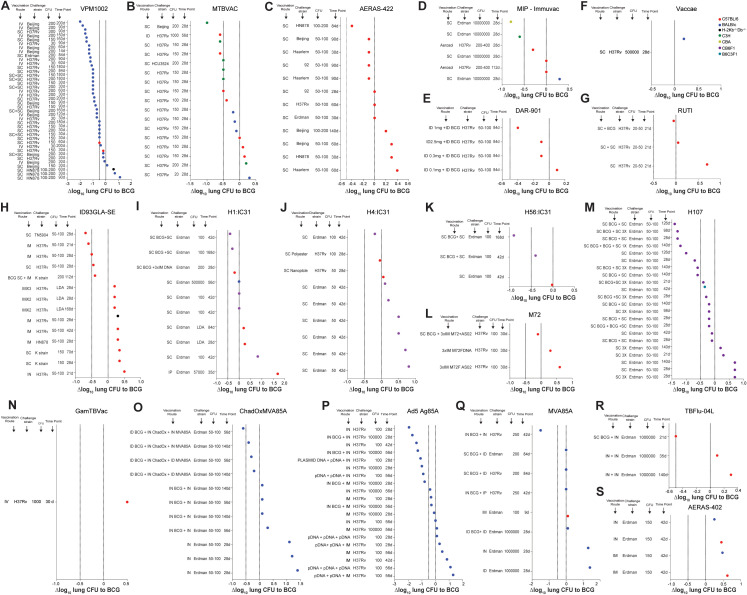
Efficacy of current and former clinical TB vaccine candidates relative to BCG vaccination in various mouse models. **(A-N)** Δlog_10_ change in lung *Mtb* CFU following vaccination with VPM1002 **(A)**, MTBVAC **(B)**, AERAS-422 **(C)**, MIP-Immuvac **(D)**, DAR-901 **(E)**, Vaccae **(F)** RUTI **(G)**, ID93GLA-SE **(H)**, H1:IC31 **(I)**, H4:IC31 **(J)**, H56:IC31 **(K)**, M72 **(L)**, H107 **(M)**, GamTBVac **(N)**, ChadOxMVA85A **(O)**, Ad5 Ag85A **(P)**, MVA85A **(Q)**, TBFlu-04L **(R)** and AERAS-402 **(S)** in various vaccination route - *Mtb* strain - *Mtb* dose - timepoint combinations compared to BCG vaccinated controls. Symbol color indicates mouse strain used (red; C57BL/6), (blue; BALB/C), (green; C3H), (turquoise; B6C3F1), (olive; CBA), (purple; CB6F1), (black; H-2Kb^-/-^Db^-/-^). Dotted lines indicate Δlog_10_ reference values of 0 and -0.5.

**Figure 4:**
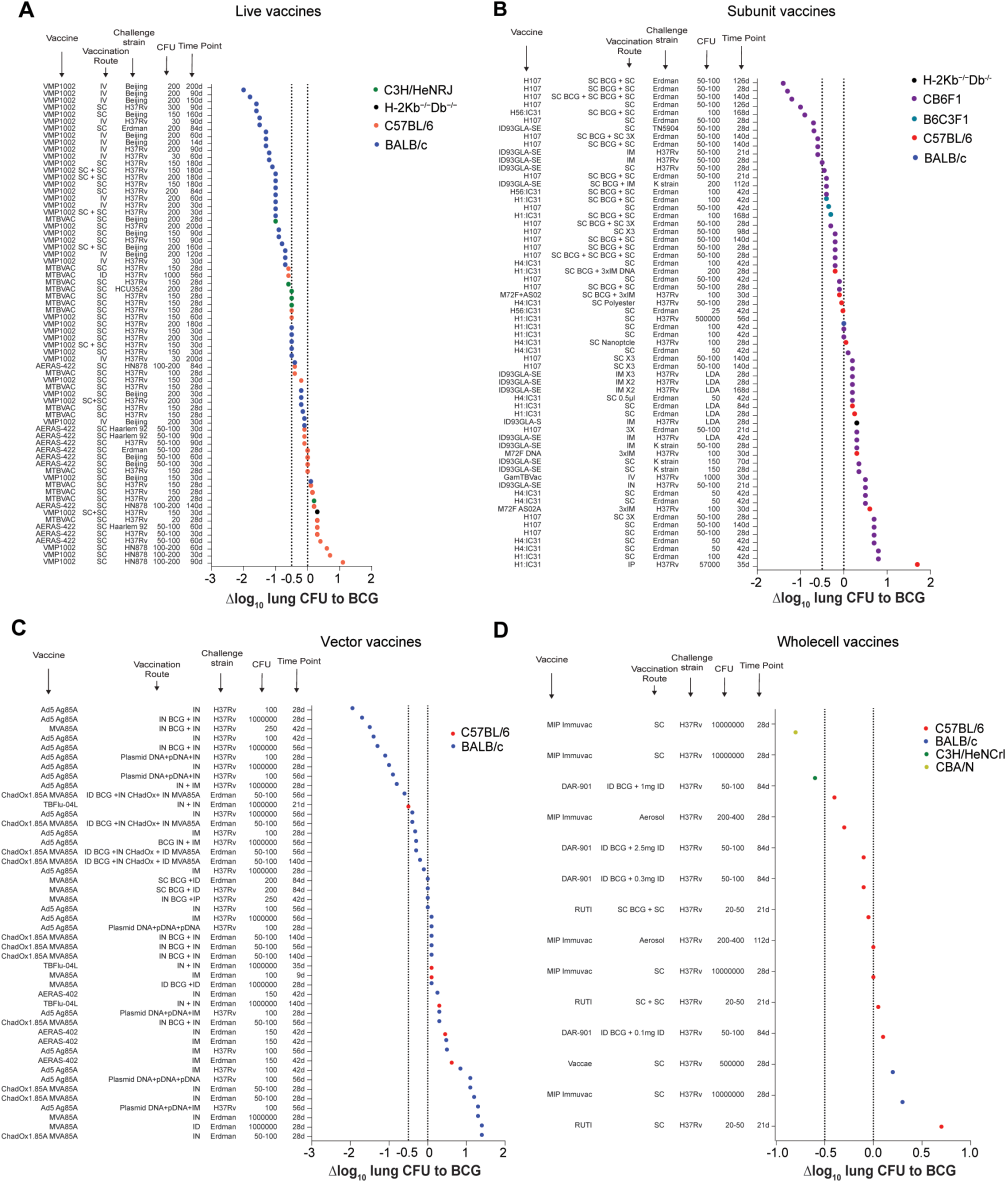
Efficacy of TB vaccine candidates relative to BCG vaccination grouped by vaccine type. **(A-D)** Δlog_10_ change in lung *Mtb* CFU following vaccination with different live attenuated **(A)**, subunit **(B)**, vectored **(C)**, or whole cell **(D)** vaccine candidates in various vaccination route - *Mtb* strain - *Mtb* dose - timepoint combinations compared to BCG vaccinated controls. Symbol color indicates mouse strain used (red; C57BL/6), (blue; BALB/C), (green; C3H), (turquoise; B6C3F1), (olive; CBA), (black; H-2Kb^-/-^Db^-/-^). Dotted lines indicate Δlog_10_ reference values of 0 and -1.

The y-axes on the graphs in **Figures 2**–**4** contain information on vaccination route (e.g., subcutaneous (SC)), *Mtb* challenge strain (e.g., H37Rv), *Mtb* challenge dose (e.g., 100 cfu) and CFU measuring time-point after *Mtb* challenge (e.g., 30 days). The x-axes show Δlog_10_ lung CFU vales with negative Δlog_10_ CFU values indicating a superiority of BCG vs. unvaccinated or vaccine candidates vs. BCG. As a point of reference, Δlog_10_ CFU values of 0 and -1.0 are highlighted with dotted lines in the comparison of BCG vs. unvaccinated graphs, and Δlog_10_ values of 0 and -0.5 in the vaccine candidates vs. BCG graphs. In the BCG vs. unvaccinated graphs, early timepoints (in black) refer to CFU enumeration at fewer than 60 days after challenge, while late timepoints (in red) indicate sacrifice after more than 60 days post-challenge. For BCG performance relative to unvaccinated mice, different studies reporting identical readout combinations, were pooled (e.g., SC vaccination + H37Rv challenge at a 30-day time-point); differences in *Mtb* challenge dose and BCG strain were not considered. The timing of the booster vaccination relative to BCG priming varies across studies, with different schedules evaluated. Please refer to the original references listed in **Table 1**; **Supplementary Table S1** for details.

The *Mtb* strains, H37Rv and Erdman were considered laboratory strains, while other *Mtb* strains (e.g. Beijing, K strain, HN878, HCU3524, SA161, CDC151, Haarlem 92) were classified as clinical isolates (**Table 2**). In the majority of studies, *Mtb* challenge was performed via the aerosol route, with specifics being detailed in **Supplementary Table S1**.

**Table 2:** *Mtb* strains, type, lineage, origin and characteristics (38, 46, 94–110).

*Mtb* Strain	Type	Lineage	Origin	Characteristics	References
H37Rv	Laboratory	4	H37Rv was first isolated from a patient (H37) with pulmonary tuberculosis in 1905 at the Trudeau Sanatorium in Saranac Lake, New York.	Reference strain, fully sequenced, widely used in research, virulent in mice	(94, 105)
Erdman	Laboratory	4	Erdman was isolated from human sputum by William H. Feldman in 1945, at Mayo Clinic, Rochester.	Virulent, higher pathogenicity than H37Rv, used in virulence and immunization studies	(94, 106)
Beijing	Clinical	2	Beijing, China	Highly transmissible, associated with drug resistance, wide geographical distribution	(99)
K strain	Clinical	2	South Korea	Hypervirulant, often used in transmission and virulence studies, drug resistant	(100, 107)
HN878	Clinical	2	Houston, USA	Hypervirulent, rapid growth	(101, 108)
HCU3524	Clinical	3	East African-Indian	Low Bacterial burden in mice	(46, 109)
SA161	Clinical	2	Arkansas, USA	Hypoimmunogenic and hypervirulent	(38, 110)
CDC1551	Clinical	4	Tennessee, USA	Highly contagious, drug sensitive	(102)
Haarlem	Clinical	4	Haarlem, Netherlands	Ubiquotous worldwide. Moderate transmissibility and virulence, long survival index.	(103, 104)

## Results

### BCG efficacy is more dependent on host genetic background than vaccination route

We first reviewed the protective efficacy of BCG against unvaccinated mice following *Mtb* infection. BCG efficacy has been evaluated in 13 different mouse strains, with the largest number of studies having been conducted in C57BL/6 and BALB/c mice, followed by C3H and CB6F1 hybrid mice (**Figure 2A**). This is also reflected in the diversity of vaccination routes. While data on all vaccination routes (SC, IT, IN, ID, IV) exists for C57BL/6 and BALB/c mice (**Figures 2B, C**), many of the other mouse strains have so-far only been vaccinated via one or two different routes.

Overall, of the 65 published mouse-route-strain-time combinations, 64 are protective, except the 129/S2-SC-H37Rv-Late combination. While, the level of efficacy varies depending on all 4 parameters, on average across all mouse strains BCG vaccination provides approximately between -0.5 and -1.0 Δlog_10_ CFU reduction compared to unvaccinated controls, with Δlog_10_ CFU values ranging from -0.1 to -2.7 (**Figure 2A**).

With few exceptions, BCG provides greater protection in all mice strains at early timepoints (<60 days, black symbols) compared to late timepoints (>60 days, red symbols). Across mouse strains, IV and IN vaccination seem to provide the best protection at early timepoints, albeit sometimes with only a relatively modest improvement over SC/ID vaccination. It also appears that the challenge strain of *Mtb* has some influence on the outcome of the protective efficacy.

The overall most protective combinations are: IV vaccination with H37Rv/Erdman challenge in C57BL/6 mice, ID vaccination with H37Rv/Erdman challenge in CB6F1 mice, SC vaccination with clinical *Mtb* challenge in C3H mice and IN vaccination with H37Rv/Erdman challenge in DBA/2 mice, which provide a Δlog_10_ CFU reduction of -1.6, -1.5 -1.8 and -1.9 respectively. At early time points, IN, IT, and IV vaccination in DBA/2, C3H, and C57BL/6 mice, respectively, provides the best protection against H37Rv and Erdman strains. In contrast, at later time points, SC vaccination provides the lowest protection against clinical *Mtb* strains, and at early timepoints IV vaccination shows the least protection against H37Rv or Erdman challenge in 129/S2, CBA/J, and C3H mice.

In C57BL/6 mice, as the most studied mouse strain (**Figure 2B**), BCG showed the strongest protective efficacy when administered IV, IN or IT, especially when using H37Rv or Erdman *Mtb* challenge strains at early timepoints. Intriguingly, the two least protective combinations in C57BL/6 mice were IV vaccination with a late efficacy timepoint after H37Rv/Erdman or clinical *Mtb* challenge. The more studies existed for a particular route-strain-time combination, the more likely the results clustered around a very tight Δlog_10_ CFU reduction value, indicating reproducibility of results across different laboratories. A similar trend was observed in BALB/c mice (**Figure 2C**), with the greatest protection achieved at early timepoints after IN and ID BCG administration and at late time points after IV administration of BCG. Similarly to C57BL/6 mice, the least protective combination in BALB/c mice was IV vaccination with a late efficacy timepoint after clinical *Mtb* challenge. Early timepoints in these mice generally also showed better efficacy against *Mtb* challenge, with the greatest protection achieved against clinical *Mtb* strains, when BCG was delivered IN.

Collectively, the analysis so far suggested that murine host genetics has the largest influence on BCG performance followed by time, vaccination route and *Mtb* challenge strain. To further assess these conclusions, we next plotted the Δlog_10_ lung CFU obtained in CC and DO mice (**Figure 2D**). So far, only five published studies have reported the performance of BCG vaccination in CC and DO mice. They used SC, ID and IV vaccination routes for CFU enumeration after challenge. In contrast to the results seen in different inbred mouse strains (**Figure 2A**), the Δlog_10_ CFU values in DO mice ranged from around -4.0 to +2.5 (**Figure 2D**), indicating a very heterogenous response to BCG vaccination due to host genetic variability.

In line with less genetic diversity compared to DO mice, CC mice showed a slightly tighter response variability with Δlog_10_ CFU values ranging from -1.8 to +0.8. To allow objective interpretation of variability in BCG efficacy, we calculated the statistical range and variance across vaccination routes, mouse strains, *Mtb* challenge strains and time points (**Supplementary Table S2**). While the range of responses in individual inbred mouse strains across all parameters only showed a range from 0 to 2.8 with a variance of 0 to 1.47, the responses in DO and CC mice ranged from 2.48 to 6.5 with a variance of 0.41 to 2.2. These results strongly support the notion that host genetic variability has a significant influence on BCG performance in mice.

### Performance of clinical TB vaccine candidates in mouse models

Next, we extracted data from all TB vaccine candidates that have been in clinical trials between 2002 and 2024. In total, we identified 22 vaccines candidates, of which 15 are currently in clinical trials and 7 that have been in clinical trials previously (excluding BCG revaccination) (**Figure 1**). For 3 of those candidates no published data on pre-clinical efficacy evaluation in mice could be found (AEC-BC02 (111), BNT164a1 and BNT164b1) and for some vaccines only very limited data could be found. For the M72/AS01_E_ vaccine candidate, only mouse studies using M72 without the AS01_E_ adjuvant were identified. We have reviewed the performance of each candidate relative to the BCG control group.

### Live attenuated vaccines

Currently, there are two main strategies employed in developing recombinant LAVs for TB - the deletion of genes in *Mtb/*BCG and/or the introduction of additional genes into BCG. MTBVAC and VMP1002 are two examples of these types of LAVs which have progressed to different stages of clinical trials. While MTBVAC is an attenuated *Mtb* strain with deletions in *phoP* and *fadD26*, VPM1002 is a recombinant BCG strain that expresses listeriolysin and contains a deletion in *ureC*.

#### VMP1002

The performance of VPM1002 was compared to BCG in 9 mouse studies (47–54), leading to 40 individual data points (**Figure 3A**). Most of the studies, except one, have been performed with BALB/c mice, with 36/40 route-strain-dose-time combinations showing enhanced protection against *Mtb* with Δlog_10_ values lower than BCG. Interestingly, many of the most protective results were obtained when VPM1002 was administered IV and CFU load was enumerated at late time points after challenge. While the *Mtb* challenge dose had relatively little impact on the overall protection, it appears that the lowest level of protection was seen in mice that were sacrificed at early time points. For C57BL/6 mice, only 2 data points are available, but the results are similar, with earlier time points (30 days post-challenge) showing lower protective efficacy compared to later time points (60 days post challenge). BALB/c mice showed greater CFU reductions across a range of conditions, particularly when IV vaccination and a Beijing challenge strain were used. SC and SC+SC (prime-boost) administrations also demonstrated strong efficacy, though generally to a lesser degree than IV. *Mtb* H37Rv, Erdman, HN878 and Beijing strains were used for challenges. The largest degree of protection was seen in BALB/c mice following IV vaccination and *Mtb* Beijing challenge with 200 CFU at 90 and 200 days after challenge.

#### MTBVAC

MTBVAC has been compared to BCG in 6 mouse studies (41–46) with 17 individual data points (**Figure 3B**), with protection ranging from Δlog_10_ CFU values of -1.0 to +0.3. In 12/17 readouts MTBVAC was superior to BCG, in 1/17 readouts equivalent to BCG and in 4/17 readouts inferior to BCG. Apart from one study, MTBVAC has been used via SC vaccination across three different mouse strains (C57BL/6, BALB/c, and C3H/HeNRJ), with all of those having enumerated CFU values at 28 days post *Mtb* challenge.

The largest protective efficacy of a Δlog_10_ CFU value approaching -1.0 was observed in C3H/HeNRJ mice vaccinated SC and challenged with *Mtb* Beijing. In other conditions, SC vaccination using BALB/c and C57BL/6 generally resulted in approximately -0.5 to 0 Δlog_10_ CFU values compared to BCG.

ID vaccination with MTBVAC in C57BL/6 followed by a at a high dose (1000 CFU) challenge with H37Rv and a CFU readout at 56 days after infection led to a reduction in lung CFU relative to BCG of -0.6 Δlog_10_.

#### AERAS-422

The efficacy of BCG against AERAS-422 has been evaluated in three mice studies (54–56) with 12 individual data points (**Figure 3C**), using C57BL/6 mice and SC vaccination route, different challenge strains (H37Rv, Beijing, HN878 and Erdman), challenge doses (50–100 and 100–200 CFU) and CFU enumeration timepoints (30, 60, 84, 90 and 140 days). SC vaccination with a high dose challenge with *Mtb* HN878 showed an enhanced protection over BCG, with a Δlog_10_ value close to -0.4. In contrast, SC vaccination with a 50–100 cfu challenge with *Mtb* Haarlem, H37Rv, Beijing or HN878 at early timepoints (30 and 60 days) generally showed no improved protection over BCG with Δlog_10_ values at or below zero.

### Whole cell vaccines

While several inactivated/killed whole cell mycobacterial formulations have been developed to be used as therapeutic vaccines/immunotherapies in people with TB, some of them have also been evaluated in their capacity to prevent or reduce TB in mouse models. These include DAR-901, *Mycobacterium indicus pranii* (MIP), RUTI, *Mycobacterium vaccae* and SRL172.

#### MIP-Immuvac

The efficacy of BCG against MIP-Immuvac (**Figure 3D**), has been evaluated in 2 published mouse studies (59, 60) with 6 individual data points using different mouse strains (C57BL/6, BALB/c, CBA, C3H/HeNRJ), vaccination routes (SC and aerosol), challenge strains (H37Rv and Erdman), challenge doses (200 and 10,000,000 CFU) and CFU enumeration timepoints (28 and 12 days). SC vaccination with a high dose challenge with *Mtb* Erdman (10,000,000 CFU) in CBA and C3H/HeNRJ mice showed an enhanced protection over BCG, with a Δlog_10_ value close to -0.8 in CBA mice and a Δlog_10_ value close to -0.6 in C3H/HeNRJ mice.

In contrast, SC or aerosol vaccination in C57BL/6 mice showed no or minimally improved protection over BCG with Δlog_10_ values at or slightly below zero. SC vaccination in BALB/c mice followed by a high dose Erdman challenge was inferior to BCG with a Δlog_10_ of +0.3.

#### *M. vaccae/*SRL172/DAR-901

Injectable killed *Mycobacterium vaccae* (Vaccae™) has been approved in China as adjunct immunotherapy to TB treatment in 2001 (112). Another heat-killed preparation of non-tuberculous mycobacteria, initially thought to be *Mycobacterium vaccae*, (**Figure 3F**), but later identified to be *Mycobacterium obuense* (57, 113) is known as SRL172. It has been tested in one study in BALB/c mice in a study by Liu et al. in 2015 (58).

Subsequently, DAR-901 (*M. obuense*) (**Figure 3E**), was produced via a scalable broth-based manufacturing method from the same master stock as SRL172 (114). The protective efficacy of vaccination with DAR-901 was measured by Lahey et al. in 2016 (57) using ID vaccination of 4 different doses in conjunction with BCG in C57BL/6 mice, a challenge with *Mtb* H37Rv (50–100 CFU) and a CFU readout at 84 days post-infection. In this study, 1mg of DAR-901 in combination with BCG provided the best protection, with a reduction in lung CFU Δlog_10_ value approaching -0.4 relative to BCG alone. It appears that a higher dose does not correlate with greater protection, because a 2.5mg dose of DAR-901 provided similar protection to a dose of 0.3mg, which was only slightly better than BCG alone (-0.1 CFU Δlog_10_ reduction). The lowest dose of 0.1mg provided no additional benefit over BCG with a Δlog_10_ value of +0.1.

#### RUTI

Preventative vaccination with RUTI has only been tested in one mouse study performed in C57BL/6 mice by Vilaplana et al (61). When administered SC, RUTI vaccination (**Figure 3G**), doesn’t appear to provide improved protection against *Mtb* H37Rv challenge compared to BCG. When RUTI is combined with SC BCG vaccination, it shows a slight improvement in protection over BCG alone with a Δlog_10_ value of -0.05.

### Subunit vaccines

Several subunit vaccines consisting of mycobacterial antigens combined with different adjuvants have been or currently are being evaluated in clinical trials. Six of those vaccine candidates have been compared to BCG in pre-clinical mouse studies.

#### ID93GLA-SE

The efficacy of ID93GLA-SE compared to BCG (**Figure 3H**), was evaluated under various conditions in 8 studies (62–69), including different routes of administration, and *Mtb* challenge strains resulting in 14 individual data points. Most of the data analyzed involved C57BL/6 mice. In 5/14 conditions ID93GLA-SE was superior to BCG. The protective efficacy varies with different *Mtb* challenge strains and vaccination routes, and the best protection in C57BL/6 mice seems to be provided when the vaccine is administered IM or SC and efficacy is measured at early time points using the H37Rv and TN5904 *Mtb* strain with Δlog_10_ values of -0.5 or better. Vaccinations in H-2Kb^−/−^Db^−/−^ mice, the use of the *Mtb* K or H878 strains, and repeated intramuscular vaccination (e.g., IMx2 or IMx3) followed by low-dose administration (no exact CFU values given) challenge showed weaker protection, with positive Δlog_10_ values.

#### H1:IC31

Six studies assessed the protective capacity of H1:IC31 compared to BCG in three different mouse strains (C57BL/6, BALB/c, CB6F1) (71–78) leading to 10 individual data points (**Figure 3I**). A SC booster of previous BCG vaccination with H1:IC31 in CB6F1 mice led to superior protection at early and late timepoints. Administration of H1:IC31 by itself does not seem to provide better efficacy than BCG regardless of the mouse strains, administration route, the challenge dose or the timepoint after infection.

#### H4:IC31

The protective capacity of H4:IC31 (**Figure 3J**) was evaluated across 3 studies in CB6F1 and C57BL/6 mice involving 9 individual data points (72, 79, 80). A slightly improved protection was reported when H4:IC31 was administered SC followed by a 100 CFU *Mtb* Erdman challenge and protection was evaluated 42 days post challenge in CB6F1 mice (72). In other studies, the use of different doses and formulations of H4:IC31 did not provide improved protection compared to BCG (79, 80).

#### H56:IC31

H56:IC31 (**Figure 3K**) was evaluated for protective capacity in two studies with a total of 3 individual data points (76, 81). In all 3 conditions H56:IC31 was superior to BCG. The largest improvement in protection was observed at a late timepoint (168 days post challenge) when H56:IC31 was used in a prime-boost strategy together with BCG in CB6F1 mice (76). In contrast, H56:IC31 vaccination by itself in C57BL/6 provided only a Δlog_10_ CFU reduction of -0.02 compared to BCG at 42 days post-challenge.

#### H107

The efficacy of H107 (**Figure 3M**), was compared to BCG in 2 mouse studies leading to 22 individual data points (82, 83). As one of the more recent vaccine candidates to enter the clinical trial pipeline, H107 has been evaluated in F1 crosses of C56BL/6 mice with BALB/c mice (CB6F1) as well as F1 crosses of C57BL/6 mice with C3H/HeNRJ mice (B6C3F1). In both studies, challenge occurred with 50–100 CFU of *Mtb* Erdman, and CFU enumeration was performed at various time points ranging from 21–140 days. In 17/22 conditions H107 was superior to BCG, especially when used in combination with BCG. The most effective regimens involved sequential SC BCG and SC H107 boosts, consistently showing enhanced protection over BCG with Δlog_10_ CFU reductions ranging from -0.5 to -1.5, particularly at later time points.

H107 administered alone at various time points (21, 28, and 98 days) showed modest protection compared to BCG, but did not achieve the same level of bacterial reduction observed with combined BCG + H107 regimens. Single or triple doses of H107 without BCG ranged from modest protection (near -0.5 Δlog_10_) to slight increases in CFU compared to BCG alone, particularly in B6C3F1 mice. Overall, the available data suggest that in mice H107 is most effective as a booster in conjunction with BCG.

#### M72/AS01_E_

No mouse studies on M72 with the adjuvant AS01_E_ are available, therefore the only available studies using M72 formulated with AS02 or DNA in mice have been included in this review. The efficacy of M72F (**Figure 3L**), was compared to BCG in 2 mouse studies leading to 3 individual data points (84, 85). However, in 2/3 of the tested conditions in C57BL/6 mice M72F was inferior to BCG. While vaccination with M72F alone does not seem to offer better protection compared to BCG, a BCG prime followed by 3 doses of IM M72F boost, showed a slight improvement when compared to BCG with Δlog_10_ CFU values of -0.1 at 30 days post *Mtb* H37Rv infection.

#### GamTBVac

Only one published mouse study in which GamTBVac was compared to BCG could be identified (**Figure 3N**). Tkachuk et al. (70) showed that IV GamTBVac vaccination followed by a challenge with *Mtb* H37Rv in C57BL/6 mice led to lower efficacy than BCG vaccination 30 days after *Mtb* infection.

### Vector vaccines

For 5 vector-based TB vaccine candidates pre-clinical moue studies that included a BCG control group could be identified.

#### ChadOxMVA85A

ChadOxMVA85A (**Figure 3O**) efficacy was evaluated in 2 studies using BALB/c mice with a total of 11 individual data points (86, 87). In 4/11 of the tested conditions ChadOxMVA85A was superior to BCG. While vaccination with ChadOxMVA85A alone does not seem to offer better protection compared to BCG, a BCG prime followed by an ID or IN ChadOxMVA85A boost, showed Δlog_10_ CFU values of -0.2 to -0.5 at 56 and 140 days post *Mtb* infection (86, 87).

#### Ad5Ag85A

Vaccination with Ad5Ag85A (**Figure 3P**), was evaluated for its performance in BALB/c mice in 3 studies with a total of 20 individual data points (88, 89). IN administration of the vaccine demonstrated an enhanced protective effect against *Mtb*, achieving substantial bacterial load reductions of up to Δlog_10_ CFU values of almost -2.0 either when used alone or in combination with BCG, particularly at higher challenge doses (100,000 CFU of H37Rv) and at later time points (56 days post-challenge), especially when combined with BCG (92). Some IM vaccination regimens also showed protective effects, although generally at lower Δlog_10_ CFU values than the IN route. Combinations involving plasmid DNA followed by Ad5Ag85A further improved the protection. For example, plasmid DNA + plasmid DNA + IN AdAg85A at 100 CFU H37Rv 28 days post-challenge showed strong protective effects with Δlog_10_ CFU values of -1.1 (115). Overall, it appears that Ad5Ag85A confers the greatest protection in mice at earlier timepoints when administered IN or in a plasmid DNA prime- Ad5 Ag85A boost configuration.

#### MVA85A

The efficacy of MVA85A (**Figure 3Q**) was evaluated in 4 studies in C57BL/6 and BALB/c mice with a total of 8 individual data points (53, 86, 90, 91). In 1/8 of the tested combinations MV85A was superior to BCG. In this study from Goonetilleke et al. (91), IN BCG + IN MVA85A vaccination followed by a challenge with 250 CFU *Mtb* H37Rv led to a reduction in bacterial load of approximately Δlog_10_ of -1.5 at 42 days post-challenge. MVA85A administered SC or IP, either alone or in combination with BCG, does not appear to provide better protection than BCG alone.

#### TBFLU-04L

TBFlu-04L (**Figure 3R**) was compared to BCG in 1 study (92) using C57BL/6 mice with 3 individual data points (92). In this study, administration of IN TBFlu-04L alone did not provide a protective effect compared to BCG, with a decline in efficacy between day 35 and 140. When BCG was combined with IN TBFlu-04L and mice were challenged with a high dose of *Mtb* Erdman (1,000,000 CFU), a Δlog_10_ CFU reduction of -0.5 was observed at 21 days post-challenge, indicating some enhancement in protection compared to BCG alone.

#### AERAS-402

Vaccination with AERAS-402 (**Figure 3S**), was evaluated for its performance in BALB/c and C57BL/6 mice in 1 study (93) with a total of 4 individual data points. IN and IM administration of the vaccine does not appear to provide an improved protective effect against *Mtb* Erdman (150 cfu) 42 days after challenge when compared to BCG.

### mRNA vaccines

Two mRNA vaccine candidates (BNT164a1 and BNT164b1) entered the TB vaccine pipeline in 2024 (**Figure 1**), but no published reports of preclinical mouse studies could be identified at the time when literature search was completed.

### Comparison of vaccine types

As stated above, the purpose of this article is not to compare the performance of individual vaccine candidates against each other, nor to provide any judgement about their potential value as future TB vaccines. Nevertheless, we have also plotted all data points from each vaccine type (LAV, whole cell, subunit, vectored) to provide an overview on how individual results rank across multiple vaccine candidates (**Figure 4**).

### Live vaccines

In 52/69 conditions tested, MTBVAC, VMP1002 and AERAS-422 provided a larger lung CFU reduction than BCG, with 39/69 showing Δlog_10_ CFU values of -0.5 or below (**Figure 4A**). Out of the top 25 conditions, 21 have been measured at late time points (≥60 days); 11/25 were derived from studies in which the LAV was given IV; 10/25 were from challenges with *Mtb* Beijing; and 24/25 data points were derived from BALB/c mice. The most protective regimen achieving a Δlog_10_ CFU of -2.0 was obtained with an IV vaccination with VPM1002 in BALB/c mice at 200 days after a challenge with *Mtb* Beijing. From the bottom 25 conditions, 21 were measured at early time points (mainly 30 days and below); 24/25 were obtained after SC vaccination; 5/25 were from challenges with *Mtb* Beijing; and 1/25 were from C3H/HeNRJ mice, 17/25 from C57BL/6 mice, 1/25 from H-2Kb^-/-^Db^-/-^ mice and 6/25 from BALB/c mice.

Collectively, these data suggest that LAVs predominantly provide superior protection over BCG, and that this protection is most pronounced at late time points and following IV and SC prime-boost administration in BALB/c mice.

### Subunit vaccines

In 30/62 conditions subunit vaccines provided greater protection than BCG, with 11/58 showing Δlog_10_ CFU values of -0.5 or below (**Figure 4B**). The overall assessment highlights the fact that subunit vaccines may provide the greatest efficacy in mice when given in combination with BCG. In 16 of the top 25 conditions the subunit vaccine candidate was given as a booster; 9/25 were recorded at late time points; 21/25 were derived from CB6F1 hybrid mice; and 14/25 resulted from vaccinations with H107. The most protective regimen achieving a Δlog_10_ CFU of -1.5 was obtained with a SC boost of BCG with H107 in CB6F1 mice at 126 days after a challenge with *Mtb* Erdman. The 25 least protective regimens exclusively include conditions without BCG prime and all of them were inferior to BCG vaccination alone. In 21/25 conditions the CFU results were recorded at early time points; 3/25 were measured after challenge with a clinical *Mtb* isolate; and 7/25 were in C57BL/6, B6C3F1 and H-2Kb^-/-^Db^-/-^ mice. Collectively, these data suggest that subunit TB vaccine candidates predominantly provide superior protection over BCG when given to hybrid mice as a boost to a BCG prime vaccination.

### Vector vaccines

The efficacy of vector vaccines was superior to BCG in 18/46 tested conditions, with 11/46 providing improvements of Δlog_10_ CFU of -0.5 or below (**Figure 4C**). In 24/25 of the top 25 conditions, efficacy was tested in BALB/c mice; 18/25 involved some form of IN delivery of the vector vaccine or a BCG prime; and 22/25 results were obtained at early time points. The most protective regimen with a Δlog_10_ CFU value of -1.947 was IN Ad5 Ag85A vaccination in BALB/c mice, followed by a challenge with *Mtb* H37Rv and CFU readouts obtained at 28 days after infection.

When the bottom 25 regimens are assessed, only 1/25 conditions provide similar protection over BCG; and 24/25 are inferior to BCG vaccination. In 22 of the bottom 25 regimes CFU readouts were measured at early time points, and none of them were tested against a clinical *Mtb* strain.

Collectively, these data suggest that vectored TB vaccine candidates can provide superior protection to BCG either when administered alone or in combination with BCG, particularly, following IN vaccine delivery.

### Whole cell vaccines

In total only 14 data points comparing whole cell TB vaccine candidates to BCG have been identified (**Figure 4D**). In 7/14 conditions whole cell vaccines showed superior protection than BCG, albeit at a relatively low level compared to other vaccine candidates. In 2/14 conditions whole cell vaccines showed equal protective efficacy to BCG and in 5/14 conditions BCG vaccination was superior. The most protective regimen achieving a Δlog_10_ CFU of -0.8 was obtained with an SC vaccination with MIP-Immuvac in CBA mice at 28 days after a high dose challenge with *Mtb* H37Rv. The second most protective vaccination regimen was demonstrated in C3H/HeNCrl mice. Inferior regimens included SC and ID vaccinations with RUTI, DAR-901 and MIP-Immuvac following both high- and low dose challenges at early and late time points. Compared to the other vaccine types, whole cell vaccines show the least clear trend across genotype, time and vaccination route regarding what conditions may influence vaccine efficacy in mice the most.

## Conclusions

There remains strong debate in the TB vaccine research community about the *ideal* mouse model to evaluate and shortlist novel vaccine candidates. Our systematic analysis of the existing literature on efficacy of clinical TB vaccine candidates in mice further supports that i) host genetics plays an important role in determining the efficacy of BCG in mice (38, 116); and ii) that vaccine type, vaccination route, and the duration of *Mtb* challenge influence the protection levels achievable with TB vaccine candidates in mice. The analysis also suggests that *Mtb* challenge dose (ranged from 1 – 10,000,000 CFU) only has a minor impact on vaccine efficacy.

While we do not intend to make any judgements on the translational potential of any of the vaccine candidates reviewed in this paper, it is clear that the performance of most TB vaccine candidates differs amongst mouse strains, administration routes and dosing regimens. As such, it will likely remain necessary to perform vaccination-challenge studies in a set of different mouse strains spanning the spectrum of susceptibility to obtain robust data for an informed decision to progress a vaccine candidate along the developmental pipeline. Among the different vaccine types, LAVs (particularly VPM1002 and MTBVAC) show significant and consistent superior protection compared to BCG under most conditions. While IV administration in BALB/c mice was most favorable for VPM1002, it is important to note that BALB/c mice were overrepresented in the VPM1002 studies, and that IV vaccination has not been evaluated for MTBVAC and AERAS-422. In the subunit category, H107 and ID93GLA-SE demonstrate greater protection than BCG, when administered as a booster to or in combination with BCG, especially in CB6F1 mice. Vector vaccines such as ChadOxMVA85A and Ad5Ag85A also provide strong protection, particularly when used in combination with BCG, as IN delivery or via multiple boosts. Whole cell vaccines offered relatively limited improvements over BCG compared to the other vaccine types, which may not be surprising as whole cell vaccines are commonly heat killed preparations that were predominantly developed for therapeutic application (113).

Our findings reinforce the importance of considering genetic diversity in murine TB vaccine studies. In none of the scenarios analyzed in this study, C57BL/6 mice showed the most pronounced protection, despite them having been used most widely in the field for decades. Particularly vaccines that have entered the vaccine development pipeline relatively early were often tested in C57BL/6 mice. Our review indicates that F1 hybrid mice may provide advantages when assessing vaccine performance, particularly for subunit and vector-based vaccines. If the community can’t agree on the *ideal* mouse model for shortlisting of vaccine candidates, DO and CC mice may provide the most valuable insights into vaccine performance across different genetic backgrounds and better mimic the variability seen in human immune responses to BCG vaccination and *Mtb* challenge (116). While the breeding of and experimental procedures in DO mice are logistically more challenging and expensive than that of inbred mouse lines, the added advantages of understanding population-wide variability to vaccination may offset those challenges. A compromise may also be the use of a few CC strains that sit at the opposite spectrum of BCG efficacy and either provide larger protective window compared to C57BL/6 mice or can’t be protected by BCG at all.

Furthermore, our review revealed that the timing of efficacy evaluation also has a substantial influence on the level of superiority over BCG that can be achieved. While early timepoints (before 60 days post-challenge) showed greater reduction in lung CFU than later timepoints across various mouse models when BCG was compared to unvaccinated mice, improved LAV protection was largely seen at later time points when BCG efficacy seems to wane, particularly against challenge with virulent clinical *Mtb* isolates. This suggests that assessing vaccine efficacy at later stages of infection may provide a better opportunity to differentiate a vaccine candidate from BCG. While our analysis also demonstrates an impact of BCG performance when administered via alternative routes, such as IN and IV, vaccination-challenge experiments in DO and CC mice argue against a very strong influence of the vaccination route compared to host genetics. Furthermore, while co-administration of subunit and vectored vaccine candidates with BCG and the use of different booster strategies appears to significantly enhance vaccine performance, LAVs may be more suitable as BCG replacement vaccines.

In summary, this study provides a useful and comprehensive comparison of the performance of all current and previous TB vaccine candidates in clinical development relative to BCG in mice, as well as the performance of BCG relative to unvaccinated mice. The intention of this review of over 200 studies was to compile available preclinical data to serve as a benchmark resource for current and future TB vaccine developers to compare the performance of their vaccine candidate, and to make informed decisions about the value of different mouse models. We would like to mention that there are also many other studies on TB vaccine candidates that are yet in preclinical rather than in clinical development. Given the large number of these studies, they have not been considered for this review paper, for logistical reasons. Furthermore, this review also highlights the broad variability of preclinical TB vaccine screening in mouse models and the challenges this variability presents when assessing future candidates. To make the most of limited research funding, it would benefit the TB vaccine research community to come to a consensus on pre-clinical screening models. Hence, we hope that this article will contribute to the debate in the community to identify (a) mouse model(s) that can be used to shortlist TB vaccine candidates in the future.
